# Impact of Elevated Low-Density Lipoprotein and the Risk of Acute Coronary Syndrome in Rheumatoid Arthritis: A Retrospective Study

**DOI:** 10.7759/cureus.80430

**Published:** 2025-03-11

**Authors:** Yousuf Sherwani, Shakir Al-Ezzi, Senthil Thambidorai

**Affiliations:** 1 Department of Internal Medicine, Medical City Arlington Hospital, Arlington, USA; 2 Department of Cardiology, Medical City Fort Worth Hospital, Fort Worth, USA

**Keywords:** low density lipoprotein-cholesterol, non-st segment elevation myocardial infarction (nstemi), retrospective cohort, rheumatoid arthritis, st elevation mi

## Abstract

Objective

To analyze the effect of hyperlipidemia on the risk of acute coronary syndrome (ACS) in patients with rheumatoid arthritis (RA).

Methods

This is a retrospective cohort study from 171 hospitals in the Hospital Corporation of America database, consisting of 1,942 individuals with RA who were retrospectively followed from the year 2020 to 2023. The primary outcome was the incidence of ACS, which included ST elevation myocardial infarction, type 1 non-ST elevation myocardial infarction, and unstable angina. The primary exposure was hyperlipidemia. Odds ratios (ORs) were obtained to ascertain the relationship between ACS (ST elevation myocardial infarction, non-ST elevation myocardial infarction type 1, and unstable angina) and hyperlipidemia (low-density lipoprotein, or LDL > 100 mg/dL).

Results

A total of 31 patients had the primary outcome of ACS. In addition, 463 patients had the primary exposure of elevated LDL (hyperlipidemia). The risk of physician-diagnosed ACS was significantly greater in participants with RA who additionally had hyperlipidemia when compared to participants who had RA without hyperlipidemia (OR: 3.88; 95% confidence interval (CI): 1.90-7.93, p < 0.01).

Conclusion

Hyperlipidemia is associated with an increased risk of ACS in patients with RA.

## Introduction

Acute coronary syndrome (ACS) is well known to be the leading cause of mortality for patients with rheumatoid arthritis (RA) [1,2]. For this specific patient population, further studies showed a tendency to have atypical presentations of ACS, along with a more severe course and relatively poorer outcomes. These worse outcomes are, in part, due to systemic inflammation, which contributes to more severe plaque build-up than the general population [3]. Furthermore, oral glucocorticoids or tumor necrosis factor-alpha (TNF-alpha) inhibitors also increase the risk of cardiovascular disease, and thus, patients with RA are more likely to be on these agents than the general population [4]. The underlying mechanism behind the association between ACS and RA is complex and multifactorial, although systemic inflammation that is caused by RA, with its respective sequelae, in addition to the traditional ACS risk factors, such as hyperlipidemia, hypertension, smoking, and diabetes, are thought to be the drivers behind the phenomenon.

There is a complex interplay between RA and lipid metabolism. Factors such as systemic inflammation, environmental factors, drug therapy, or other genetic factors cause changes in the lipid levels, as well as alterations in lipid/lipoprotein structure and function [5]. We hypothesize that hyperlipidemia in RA patients substantially increases the risk of ACS, even more than the general population with hyperlipidemia, due to the synergistic effect of hyperlipidemia coupled with the underlying inflammatory burden. Thus, controlling the systemic inflammation associated with RA, along with identification and management of hyperlipidemia, is essential to prevent ACS in patients with RA [6].

The lipid paradox theory describes the finding that low circulating levels of low-density lipoprotein (LDL) are actually associated with an increased risk of cardiovascular disease in RA. The exact mechanism as to how low levels of LDL increase the risk of cardiovascular disease remains unclear. One theory postulates that inflammation in RA causes LDL to be oxidized, and this oxidized LDL resides in the vascular endothelium, where it is not easily detectable on bloodwork. Thus, the cardiovascular burden that LDL plays is underestimated in conventional blood tests. Another theory postulates that LDL is catabolized quickly in inflammatory states such as RA. Thus, inflammation is the true culprit in increasing cardiovascular risk, and low LDL is just a byproduct of a hyper-inflammatory state. The question of the exact mechanism of action of the lipid paradox remains speculative. There have been far fewer robust studies examining the relationship between elevated LDL and the risk of cardiovascular disease in patients with RA [7]. It is still yet to be determined how the complex interplay between inflammation and lipid metabolism found in RA translates to cardiovascular risk as it pertains to hyperlipidemia. We hope to examine if hyperlipidemia increases the risk of cardiovascular disease in patients with RA.

## Materials and methods

A retrospective cohort study design was implemented to test for the association between hyperlipidemia and ACS in patients with RA. A sample of 1,942 participants was retrospectively followed from the year 2020 to 2023. The exposure of interest, hyperlipidemia, was collected by the North Texas Hospital Corporation of America database at the time of recruitment. The outcome of interest, ACS, which included ST elevation myocardial infarction, non-ST elevation myocardial infarction type 1, and unstable angina, was obtained from participant hospital inpatient records through the Hospital Corporation of America database. Bivariate analysis was conducted on the resulting data, in which the outcome of interest was ACS, and the main predictor was hyperlipidemia. Additional analyses were performed for sex, body mass index (BMI), smoking status, and type II diabetes status to evaluate the effect of these variables on the main outcome of interest. All participants recruited additionally had a diagnosis of hypertension to eliminate hypertension as a confounder. The study size was calculated by using a predicted effect size of 2.0, with a significance level of 0.05% and desired power of 80%. The incidence of ACS in patients with RA was set at 7.4 per 1,000 person years [8]. A sample size of 2,638 patients was required using these parameters to evaluate if hyperlipidemia increased the risk of ACS in patients with RA. 

Beginning in 2020, the Hospital Corporation of America enrolled approximately 1,942 participants between the ages of 30 and 80 and collected deep information on biomarkers and phenotypic information of all participants. Prospective participants completed an automated survey that asked about their lifestyle and medical history. The health of the participants is tracked through linked electronic health records. By October 2023, about 31 participants had been diagnosed with ACS. Observers were blinded when choosing participants to reduce observation bias. A sample of 1,942 participants from the Hospital Corporation of America were tested for hyperlipidemia through the use of biomarkers in the form of lipid panels. Data included LDL, high-density lipoprotein (HDL), total cholesterol, and triglyceride levels. If a participant was shown to have an LDL level over 100 mg/dL, then that participant was categorized as having hyperlipidemia.

The Hospital Corporation of America provided information on BMI, age at recruitment, sex, race, smoking status, and comorbidities. Age of recruitment was defined as the difference between the year of recruitment and the year of birth. BMI was calculated using the participant’s height and weight measured during the initial recruitment visit. Sex was defined as either male or female. Race was defined as White participants, Black participants, or other participants. Smoking status was defined as smoker or non-smoker. Finally, comorbidities were assessed using the Eixhauser Comorbidity Index. Participants have been prospectively followed since 2020 through the use of North Texas Hospital Corporation of America-linked medical records. The primary outcomes of interest were diagnoses of ST elevation myocardial infarction, non-ST elevation myocardial infarction type 1, and unstable angina. The data used in this study included the 10th Revision of the International Statistical Classification of Diseases (ICD-10) codes. Only cases of ACS confirmed through the use of electrocardiogram (EKG), troponin, and cardiac catheterization were included for analysis.

IBM SPSS Statistics for Windows, Version 24 (Released 2016; IBM Corp., Armonk, NY, USA) was used for data management, and statistical analysis systems were used for statistical analysis. Descriptive data were expressed as frequencies for categorical variables and mean ± standard deviation for continuous variables. Normal distribution for continuous variables was assessed. χ² square testing for categorical variables and t-testing for continuous variables were performed to determine significant differences (p < 0.05) between ACS and control participants. Bivariate analysis was conducted, in which the outcome variables were ST elevation myocardial infarction, non-ST elevation myocardial infarction type 1, and unstable angina, and the main predictor was hyperlipidemia. All participants, once recruited, were analyzed regardless of missing data or if they may have been lost to subsequent follow-up.

## Results

Baseline characteristics

Of 1,942 participants, we identified 31 cases of ACS. Specifically, 80% of the participants were female. Two-thirds were identified as White participants, while one-third identified as African American participants or other groups. Information on race, BMI, medical history, etc., was obtained by medical professionals from participants during their inpatient hospital stay. Additionally, 14% of the participants were smokers. About a quarter of the participants were diagnosed with hyperlipidemia (Table 1). The average LDL value for participants without hyperlipidemia was 86 mg/dL. The average LDL for participants with hyperlipidemia was 127 mg/dL. The incidence of ACS in patients without hyperlipidemia was 3.1 cases per 1,000 people per year. The incidence of ACS in patients with hyperlipidemia was 12.2 cases per 1,000 people per year.

**Table 1:** Baseline characteristics of the study population

Characteristic	No acute coronary syndrome	Acute coronary syndrome
Age at recruitment, mean (SD), y	64 (13.5)	66 (11)
BMI, mean (SD), kg/m^2^	30 (7)	28 (6)
Gender		
Female subjects	1,594	19
Male subjects	348	12
Race		
White participants	1,282	25
African American participants	408	2
Other participants	252	4
Smoker	280	9
Non-smoker	1,580	22
Type II diabetes	272	5
Nondiabetic	1,670	26
Hyperlipidemia	463	17
No hyperlipidemia	1,479	14

Bivariate analysis

**Figure 1:**
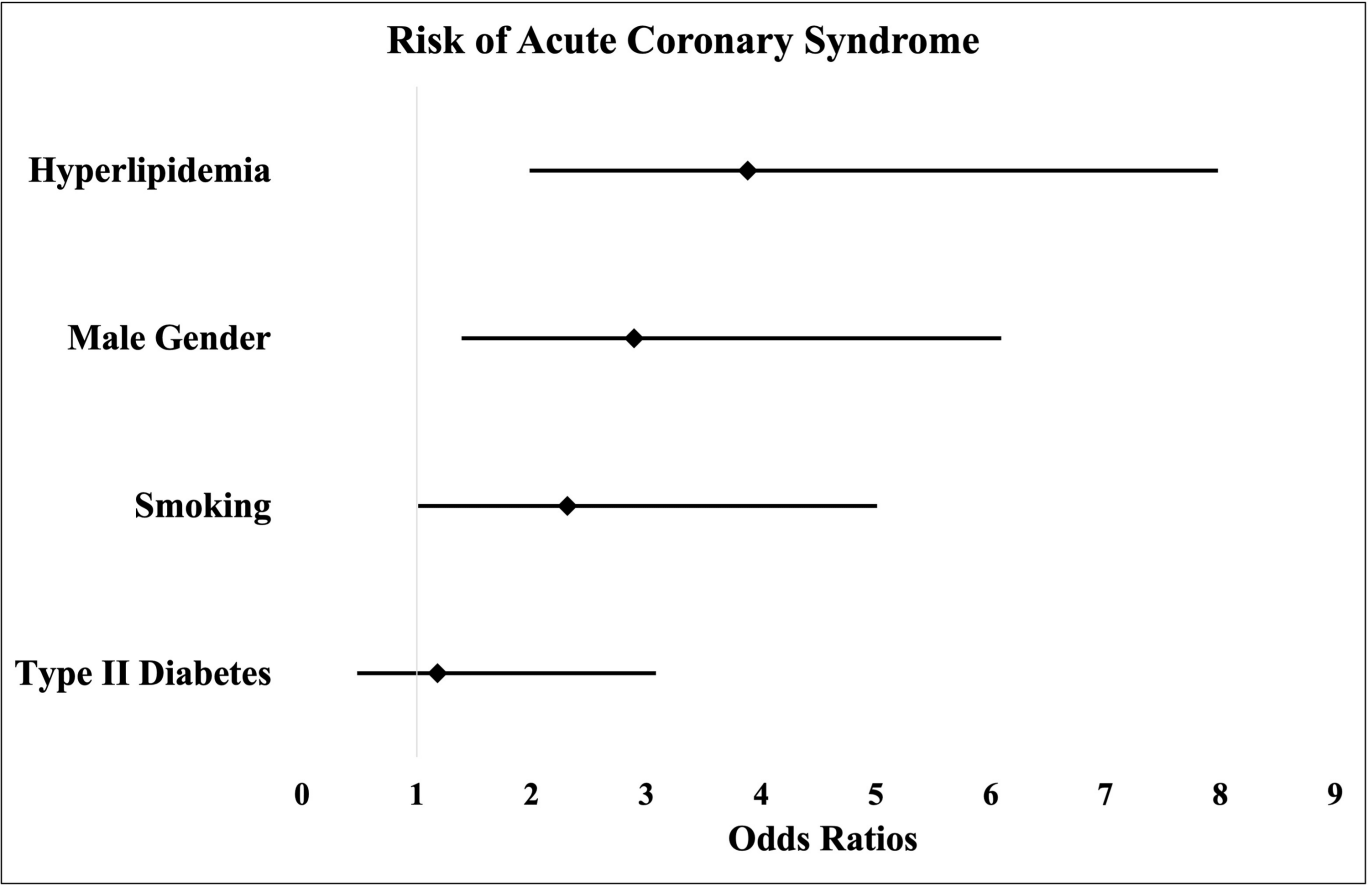
Forrest plot depicting relationship between acute coronary syndrome and rick factors Odds ratios and 95% confidence intervals for hyperlipidemia, gender, smoking status, and type II diabetes status, relating to acute coronary syndrome within the study population.

Bivariate analysis was performed to test the association between hyperlipidemia and the risk of ACS in patients with RA. After accounting for hypertension, hyperlipidemia was significantly associated with the risk of ACS (odds ratio (OR): 3.88; 95% confidence interval (CI): 1.90-7.93, p < 0.01) when compared with individuals with no hyperlipidemia (Figure 1). Additionally, hyperlipidemia was more significantly associated with an increased risk of ACS in patients with RA when compared with other classical risk factors associated with ACS (Figure 1). Other factors that increased the risk of ACS in patients with RA included male sex (OR: 2.89; 95% CI: 1.39-6.01, p < 0.01) and smoking (OR: 2.31; 95% CI: 1.05-5.06, p = 0.037). Although type II diabetes was shown to be related to an increased risk of ACS in patients with RA, this association failed to reach significance (OR: 1.18; 95% CI: 0.45-3.10, p = 0.736) (Figure 1).

To further examine the association of hyperlipidemia and ACS in patients with RA, a Chi-square analysis was conducted. The Chi-square analysis for the association between hyperlipidemia and ACS in RA was significant (Chi-square: 15, p < 0.01). The Chi-square analysis for the association between sex and ACS in RA patients was also significant (Chi-square: 8.8, p < 0.01). The Chi-square analysis for the association between type II diabetes and ACS failed to reach significance (Chi-square: 0.11, p < 0.74) (Table 2).

**Table 2:** Chi-square analysis

Characteristic	Chi-square	Significance	Cramer’s V
Gender, male	8.8	0.003	0.07
Smoker vs. nonsmoker	4.2	0.042	0.05
Type II diabetes	0.114	0.736	0.01
Hyperlipidemia	15.925	<0.001	0.09

## Discussion

In this study, hyperlipidemia was significantly associated with the risk of ACS in patients with RA (Figure 1). It is worth noting that, in our study, we controlled for hypertension by including only participants who had a previous diagnosis of hypertension from ICD-10 codes. This allowed us to control for hypertension as a confounder, as hypertension is a well-known risk factor for the development of ACS. Potential hypotheses do exist to attempt to explain the association between hyperlipidemia and the risk of ACS in patients with RA. A compelling theory is that patients with RA have increased systemic and vascular inflammation [9,10]. Patients with RA are at increased risk of developing coronary plaques compared to patients without RA due to increased vascular inflammation [11]. Hyperlipidemia and vascular inflammation are not only independently associated with atherosclerosis, but are interconnected processes [12]. Previous studies have demonstrated that hyperlipidemia and vascular inflammation have a synergistic effect in causing early atherosclerosis [13]. This enhanced atherosclerosis and plaque formation in the coronary arteries leads to an increased incidence of ACS. Our data agree with these hypotheses, as the association between hyperlipidemia and ACS in patients with RA (OR: 3.88, p < 0.01) was much more pronounced than the association between hyperlipidemia and ACS in subjects without RA (hazard ratio (HR): 1.53, p < 0.01) [14]. We postulate that appropriate treatment of hyperlipidemia in patients with RA will lead to reduced cardiovascular mortality and morbidity in this patient population. 

Growing evidence suggests that there is a complex association between hyperlipidemia and cardiovascular disease risk in RA patients [15]. For instance, in some studies, LDL and total cholesterol levels below a certain range were associated with increased cardiovascular risk in patients with RA, a phenomenon often referred to as the ‘Lipid Paradox’ [16]. Interestingly, new studies are demonstrating that RA patients with elevated LDL may also be at increased risk of developing ACS as well [17]. A promising hypothesis has been proposed that the risk of ACS and LDL levels shows a U-shaped relationship, where RA patients with low LDL and high LDL levels are at elevated risk [17]. The underlying mechanism as to why this phenomenon occurs remains speculative, and more robust studies will have to be performed to understand this phenomenon in its entirety. Our findings slightly differ from previous studies in that the risk of ACS is higher than those previously reported in the literature. These findings make a strong argument for frequent screening and education for patients with RA. At present, there are no universal guidelines for the screening and management of dyslipidemia in patients with RA. Previous studies have revealed that a large portion of patients with RA do not get screened for hyperlipidemia [18,19]. Additionally, of those patients with RA who met the criteria for treatment, only about half of those patients received said treatment [20]. This disparity, in large part, is thought to exist due to a lack of physician awareness and a lack of guideline-directed care in general [20]. Additionally, patients with RA present with atypical presentations when having episodes of ACS [3]. This further causes misdiagnosis and mismanagement of hyperlipidemia in patients with RA. Infrequent screening and treatment of hyperlipidemia in patients with hyperlipidemia leads to increased morbidity and mortality. There is a lack of guidelines concerning the management of cardiovascular disease in RA. We suggest screening for hyperlipidemia when the diagnosis of hyperlipidemia is established. Furthermore, we suggest that, when calculating cardiovascular risk score, a modifier of 1.5 be added to the calculated risk [21]. This modified RA risk score should dictate whether a patient should be started on statin medication. With the accumulation of more data, we hope that further guidelines concerning the management of hyperlipidemia in RA will be available for clinicians.

Confounders exist that could not be ruled out. Such confounders include a precise measurement of family history, accounting for genetic variance for every participant, evaluating disease-modifying antirheumatic medication, exact body composition, and assessing daily physical activity of every participant. Since data were obtained from inpatient hospitals, we were unable to assess baseline RA disease activity. This created another confounder that could not be accurately assessed due to the nature of our data. This study did not conduct multivariate analysis, so not all confounders could be controlled for. Factors such as disease-modifying antirheumatic drug (DMARD) use, steroid use, and RA disease activity levels are some of the confounders that may affect the results of our data. The actual study size was smaller than the calculated study size due to a lack of eligible participants in the database. Future large cohort studies will be needed to control for said factors by using multiple regression analysis. Due to the inclusion criteria of this study requiring hypertensive patients with RA, the results of this study cannot be generalized to RA patients without hypertension. ACS was obtained during recruitment visits between 2020 and 2023. Therefore, it may be possible that participants may have developed ACS since the end of the recruitment period. Additionally, participants may have been lost to follow-up due to moving out of the state or using other hospital networks for their care. All participants, once enrolled in the study, were analyzed to increase external validity. However, it is possible that patients who were lost to follow-up, if analyzed, may have changed the outcome of the data. As this is a retrospective study, a temporal relationship between exposure (hyperlipidemia) and outcome (ACS) cannot be ascertained with absolute certainty. It is possible that ACS in patients with RA leads to hyperlipidemia, although this seems less plausible. Additionally, in this study, causality cannot be established. A prospective study in the future will need to be performed to determine if hyperlipidemia in patients with RA causes ACS. 

## Conclusions

Heart disease remains a major problem in RA. The increased cardiovascular risk is not understood by most clinicians, due in part to the complexity of the interplay between inflammation and lipid metabolism in RA. Controlling for risk factors is an essential part of managing cardiovascular risk. Our study has shown that hyperlipidemia is a major risk factor in contributing to ACS in patients with RA. We hope to use this data to bring awareness to clinicians about the risk of ACS in patients with hyperlipidemia and RA. Future studies will help guide screening and interventional guidelines. In the meantime, we suggest clinicians be vigilant in screening for and treating hyperlipidemia in patients with RA. 
